# Assessment of the Impacts of Green Synthesized Silver Nanoparticles on *Maerua oblongifolia* Shoots under In Vitro Salt Stress

**DOI:** 10.3390/ma15144784

**Published:** 2022-07-08

**Authors:** Hassan O. Shaikhaldein, Fahad Al-Qurainy, Mohammad Nadeem, Salim Khan, Mohamed Tarroum, Abdalrhaman M. Salih, Saleh Alansi, Abdulrahman Al-Hashimi, Alanoud Alfagham, Jawaher Alkahtani

**Affiliations:** Department of Botany and Microbiology, College of Science, King Saud University, P.O. Box 2455, Riyadh 11451, Saudi Arabia; falqurainy@ksu.edu.sa (F.A.-Q.); mnadeem@ksu.edu.sa (M.N.); skhan2@ksu.edu.sa (S.K.); mtarroum@ksu.edu.sa (M.T.) abdalrahamanm@gmail.com (A.M.S.); salansi@ksu.edu.sa (S.A.); aalhashimi@ksu.edu.sa (A.A.-H.); aalfaghom@ksu.edu.sa (A.A.); jsalqahtani@ksu.edu.sa (J.A.)

**Keywords:** salt stress, silver nanoparticles, antioxidant enzymes, oxidative damage

## Abstract

Salinity is one of the major abiotic stresses that affect the plant’s growth and development. Recently, the contribution of nanoparticles (NPs) to ameliorating salinity stresses has become the new field of interest for scientists due to their special physiochemical properties in the biological system. This study is designed to examine the effects of biosynthesized silver nanoparticles (AgNPs) spherical in shape (size range between 9 and 30 nm) on morphophysiological characteristics and the antioxidant defense system of in vitro raised *Maerua oblongifolia* under four levels of salt stress (0, 50, 100, and 200 mM NaCl). Our findings reveal that the application of AgNPs (0, 10, 20, and 30 mg/L) to *M. oblongifolia* shoots significantly alleviates the adverse effects of salt stress and ameliorates plant developmental-related parameters and defense systems. High salinity elevates the oxidative damage by over-accumulation of the levels of total soluble sugars, proline, hydrogen peroxide (H_2_O_2_), and malondialdehyde (MDA). In addition, enhancing the activity of the antioxidant enzymes, total phenolic, and flavonoid content over the control. Interestingly, the application of AgNPs to salinized plants improved the growth traits and photosynthetic pigment production and caused higher enhancement in antioxidant enzyme activities. Furthermore, mitigating the oxidative damage by lowering the accumulation of proline, soluble sugars, H_2_O_2_, MDA, and total phenolic and flavonoid contents in salt-stressed plants. In general, AgNPs augmented the growth of *M. oblongifolia* shoots under saline conditions through different strategies; thus, AgNPs can be used as an appropriate eco-friendly approach that enhances salinity tolerance in plants.

## 1. Introduction

Salt stress is considered to be the most crucial abiotic factor that threatens plant distribution in their natural habitats [1]. Salinity causes adverse effects regarding plant growth, productivity, and reproduction processes, especially in arid and semi-arid zones [2]. It appears as a long list of modifications in morph-physiological and biochemical properties of plants [3]. The saline conditions have modified many physiological responses in plants, including damaging plasma membrane integrity, disrupting stomatal conductivity, producing excessive reactive oxygen species (ROS), and lowering photosynthetic efficiency [4]. Therefore, there is a continuous demand to develop new approaches to relieve the harmful impacts of these stresses on plants. Nanotechnology nowadays has gained great attention of researchers’ interest and a position in several fields of science due to the necessity of using nanoparticles (NPs) in many aspects of human needs, including industry, business, medicine, and agriculture [5]. Thus, the application of NPs is one of the new manners to ameliorate growth and plant performance under saline environments [6]. Among all types of NPs, silver nanoparticles (AgNPs) have been extensively used due to their eco-friendly implementations in biological science, unparalleled physiochemical characteristics, and large specific surfaces [7]. AgNPs increased or minimized plant growth and development depending on the concentration, size, and application time [8]. Notwithstanding, little information is accessible regarding the impact of AgNPs on plants under salty conditions [9].

*Maerua oblongifolia* (Forssk.) A. Rich., a rare medicinal plant distributed in Saudi Arabia, belongs to the family *Capparaceae*. It is used widely in the traditional herbal healing practices in Saudi Arabia to cure several diseases in both humans and domestic animals [10]. The wild populations of this plant are rapidly declining as a result of the salinity in Saudi Arabia, overexploitation for food, feed, and medicine, as well as its poor rate of regeneration [11]. Therefore, there is a serious demand to enhance the growth and morphogenesis of *M. oblongifolia* plants to adapt to the saline environment, which has considerably threatened their distribution.

The present study was made to investigate the conceivable effects of green synthesized AgNPs in the mitigation of salinity-induced oxidative stress in *Maerua oblongifolia* plants under in vitro conditions.

## 2. Materials and Methods

### 2.1. Preparation of AgNPs Using Ochradenus Arabicus

AgNPs were synthesized biologically using *Ochradenus arabicus* according to the protocol previously reported by [11]. Silver nitrate (Ag-NO_3_) was purchased from Sigma-Aldrich Chemical Corp. *Ochradenus arabicus* was obtained from the tissue culture lab of King Saud University, Riyadh. The plant extraction was performed using deionized Milli-Q water. The selected plant was cleaned and chopped into small pieces and 100 mL of distilled water was poured over it. The plant material was boiled in 100 mL water, and then cooled down at room temperature. The solution was filtered and kept in a refrigerator at 4 °C for use in further experiments. To prepare the AgNPs, 100-mL solution of 1 mM AgNO_3_ was added to the plant extract in a ratio of 1:1. The mixture was subjected to heating at 60 °C until its color changed from faint yellow to dark brown, indicating the initial AgNPs formation.

### 2.2. Characterization of Green Synthesized Silver Nanoparticles

The green synthesized AgNPs were characterized using robust techniques of analysis such as ultraviolet-visible (UV-Vis) spectroscopy, which used to verify the reduction method used for AgNPs synthesis. Fourier transform infrared spectroscopy (Termo Scientific Nicolet 6700 FT-IR spectrometer, Waltham, MA, USA) was performed to observe the presence of potential biomolecules and functional groups. For structural analysis of the synthesized silver nanoparticles, X-ray diffraction (Rigaku Ultima IV, Neu-Isenburg, Germany XRD) was used. The data were collected in 2θ range. The crystallite domain size was calculated using D. Scherrer’s equation. The particle morphology and size of the phytomediated AgNPs were photographed using transmission electron microscopy (TEM) (JEM-1011; JEOL Ltd., Tokyo, Japan).

### 2.3. Plant Growth Conditions

The plant material of *M. oblongifolia* were brought from Jazan, Saudi Arabia, and multiplied in vitro via micropropagation in Murashige and Skoog (MS) media, as per the protocol described by Al-Qurainy et al. [12].

The study was carried out in the tissue culture laboratory of King Saud University as a factorial experiment in a completely randomized design (CRD). The experiment consisted of sixteen treatments four levels of salinity (0, 50, 100, and 200 mM NaCl) as well as four levels of AgNPs (0, 10, 20, and 30 mg/L). Each treatment was performed in triplicates.

Then, *M. oblongifolia* nodal segments of 2–3 cm in length were transplanted into Magenta boxes (GA-7). Each box contained 50 mL of Murashige and Skoog (MS) medium as well as five explants. The MS media was augmented with 30% sucrose and 1.0 µM of benzylaminopurine (BA), and the pH was adjusted to 5.8. The experiment conducted in a growth chamber with controlled conditions of room temperature, light, and humidity. The temperature of the growth chamber was kept at 25 ± 2 °C, 60% RH (relative humidity and from 25 to 30 μmol m^2^ s^−1^ SFP for 16 h photoperiod day and 8 h night cycle. Samples were harvested after 45 days of culture for further analyses.

### 2.4. Growth Parameters

Plant in vitro growth performance was measured. These traits are shoot length and number of shoots per explant as measured by meter tape and counting. Besides, fresh weight of plants was recorded immediately after deflasking (taking out the plantlets from the flask). Followed by drying the samples at 105 °C in an oven day night, and dry weight of the plants was then recorded.

### 2.5. Photosynthetic Pigments 

According to Arnon [13] methodology, contents of photosynthetic pigments (chlorophyll a and chlorophyll b) were determined spectrophotometrically. In total, 0.1 g of fresh leaves were homogenized in cooled 80% acetone. The samples were stored at −4 °C for 24 h prior extraction process, and then the blend was placed in 2 mL microcentrifuge tubes. Finally, absorbance between 645 and 663 nm was assessed using a UV-1800 spectrophotometer (Shimadzu, Kyoto, Japan), with acetone being used as blank.

### 2.6. Estimation of Total Soluble Sugar and Proline Contents

Soluble sugars were evaluated using the phenol sulfuric acid method [14]. In this method, 0.3 g of plant tissue was extracted with ethanol (10 mL). The extract was centrifuged at 10,000 rpm for 5 min and then treated with 5% phenol and 98% sulfuric acid. This mixture remained for half an hour and then the absorbance was read at 490 nm using UV-Vis spectroscopy. Concentration of the total soluble sugars was determined sing a glucose standard curve.

Proline concentration was estimated following the Bates et al. [12] method. Fresh leaves (0.4 g) were crushed and 10 mL of 3% sulfosalicylic acid was added. Subsequently, the mixture was centrifuged; 2 mL of supernatant was placed into a test tube, and 2 mL of each ninhydrin reagent and glacial acetic acid were added. Afterward, the mixture was placed in a water path at 100 °C for 1 h. Following heating, the mixture was cooled in an ice bath for 5 min. Then, 6 mL of toluene to each tube was added and strenuously mixed. The absorbance of the upper phase at 520 nm was quantified using a UV-Vis spectroscopy and toluene was used as a blank.

### 2.7. Lipid Peroxidation and Hydrogen Peroxide 

Malondialdehyde (MDA) contents represent the lipid peroxidation, which was analyzed by quantifying the production of thiobarbituric acid reactive substances (TBARS) using TBARS assay. The MDA concentration in fresh tissues were analyzed following De Vos, et al. [15] Method. Leaf tissue (0.4 g) was homogenized in 5 mL of 0.1%TCA and the material was centrifuged at 10,000 rpm for 5 min. Afterward, 1 mL of clear supernatant was collected and placed in a clean test tube, to which 4 mL of 0.5% thiobarbituric acid (TBA, in 20% TCA) was added, and the blend was placed in a water bath at 95 °C 30 min. Then, the mixture was cooled in an ice bath and centrifuged at 5000 rpm for 5 min; the supernatant absorbance (containing MDA) was recorded at 532 nm and corrected to non-specific turbidity by subtracting the value at 600 nm on a UV–1800 spectrophotometer (Shimadzu, Kyoto, Japan). The blank was 0.5% TBA in 20% (*w*/*v*) TCA.

Hydrogen peroxide (H_2_O_2_) concentrations were estimated as per Velikova et al. [16] method. Fresh leaves (0.4 g) were homogenized in 5 mL of 0.1% (*w*/*v*) trichloroacetic acid (TCA). The homogenate was subjected to centrifugation at 10,000 g for 20 min; thereafter, 0.5 mL of the upper phase was mixed with 0.5 mL of 10 mM potassium phosphate buffer (pH 7.2) and 1.0 mL of 1.0 M potassium iodide solution. The absorbance was read at 390 nm spectrophotometry. 

### 2.8. Measurement of Antioxidant Enzyme Activities

Enzyme extraction and estimation were applied as per the method reported by Jogeswar, et al. [14]. Fresh leaf tissues were homogenized in 100 mM sodium phosphate buffer (pH 7.4) that contained 0.1 mM ethylenediaminetetraacetic acid, 1% (*w*/*v*) polyvinylpyrrolidone, and 0.5% (*v*/*v*) Triton-X 100. The mixture was subjected to centrifugation for 10 min at 10,000 rpm and the supernatant was then taken.

The catalase (CAT, EC 1.11.1.6) activity was quantified according to the procedure described by Greenwald [17]. In total, 1 mL of 0.059 M H_2_O_2_ in 0.1 M sodium phosphate buffer (pH 7.4), 1.9 mL of distilled water, and 100 L of enzyme extract made up the reaction mixture. The absorbance was read at 240 nm, and U/g of protein expresses the activity of CAT.

Superoxide dismutase (SOD, EC 1.15.1.1) activity was examined by following the procedure reported in Marklund and Marklund [15]. In total, 1 mL of 0.25 mM pyrogallol, 1.9 mL of 0.1 M sodium phosphate buffer (pH 7.4), and 100 L of enzyme extract made up the reaction mixture. At 420 nm, the absorbance was measured. The SOD activity (U/g protein) was defined as the amount of enzyme required for 50% inhibition of pyrogallol oxidation.

### 2.9. Estimation of Nonenzymatic Antioxidants

The Folin–Ciocalteu reagent was used to measure the total phenolic content (TPC) in accordance with the procedure described by Velioglu, et al. [18]. Fresh leaves (0.1 g) were homogenized in 99% Ethanol. The samples were shaken for 24 h at 200 rpm in an orbital shaker. After five minutes of centrifuging the mixture at 10,000 rpm, the supernatant was transferred to a 2 mL Eppendorf tube. Subsequently, 100 μL plant extract was transferred into a test tube and 0.75 mL of Folin–Ciocalteu reagent was added and thoroughly mixed in it. The mixture was placed at room temperature for 8 min. Then, 0.75 mL of Na_2_CO_3_ was incubated with the mixture at room temperature for 30 min. The absorbance was measured at 765 nm via a UV-1800 spectrophotometer (Shimadzu, Kyoto, Japan). A standard calibration curve was plotted using Gallic acid and TPC was expressed in Gallic acid equivalent. For total flavonoids content (TFC), 20 μL plant extract, 10 μL aluminum chloride, 10 μL potassium acetate, and 160 μL di-ionized water were mixed and then incubated for a quarter of an hour. The absorbance was monitored at 420 nm using a (UV-Vis) spectroscopy. A standard calibration curve was plotted using Gallic acid.

### 2.10. Statistical Analyses

Statistical analyses were performed using two-way ANOVA and mean separation between treatments was obtained using Duncan’s new multiple range test (*p* ≤ 0.05) in SPSS v. 20 for Windows.

## 3. Results

### 3.1. Characterization of AgNPs Nanoparticles

**Figure 1:**
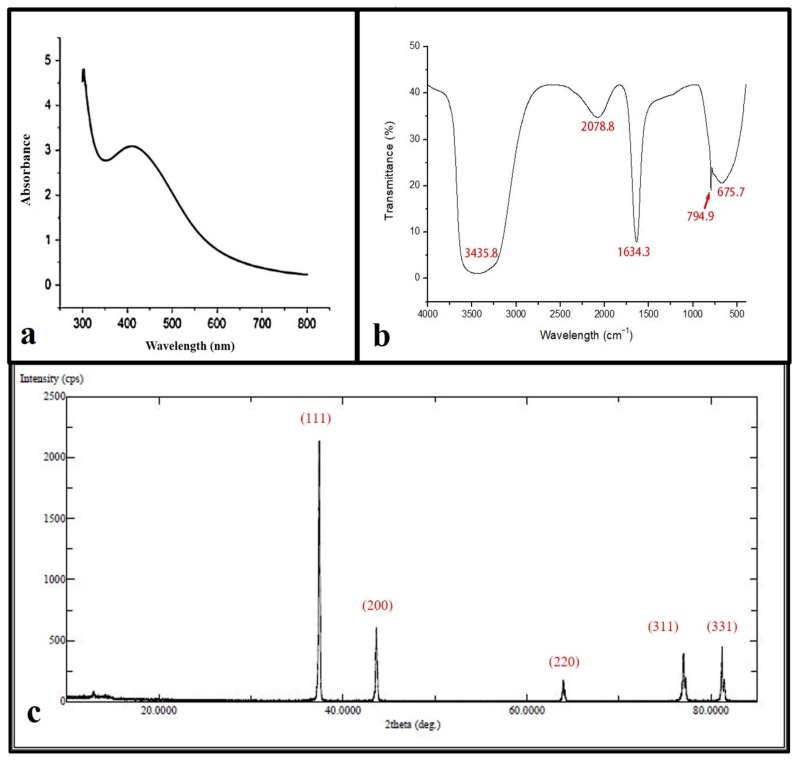
Ultraviolet-visible absorption spectrum of the silver nanoparticles (AgNPs) with a plasmon band at 400 nm (**a**). FT-IR spectrum of synthesized AgNPs (**b**). XRD patterns (**c**).

The reacted mixture of *Ochradenus arabicus* leaf extract and AgNO_3_ solution showed a color change in the extract from light yellow to brown after adding 1 mM AgNO_3_. This change in color could be an initial indicator of AgNPs formation. However, for further investigations, a UV-Vis spectrophotometer was performed to detect surface plasmon resonance (SPR) of the silver ion. The AgNPs SPR band demonstrated a characteristic peak at 400 nm, as shown in Figure 1a.

FTIR spectroscopy was used in order to detect the functional groups involved in AgNPs synthesis. The FT-IR spectrum of AgNPs ranges between 400 and 4000 cm^−1^ as shown in (Figure 1b), where the FT-IR profile demonstrated five peaks located at about 675 cm^−1^, 794 cm^−1^, 1634 cm^−1^, 2078 cm^−1^, and 3435 cm^−1^. The apparent bands are representative of functional groups of various compounds. 

XRD spectra deliver insight into the crystallinity of nanoparticles. The XRD spectrum of silver nanoparticles confirmed that the silver nanoparticles formed were in the form of nanocrystals, as evidenced by the peaks at 2θ values corresponding to (111), (200), (331), (241), and (311) Bragg reflections of silver, respectively (Figure 1c). The crystallite size of silver nanoparticles was found to be 16 nm using the Scherrer equation. 

**Figure 2:**
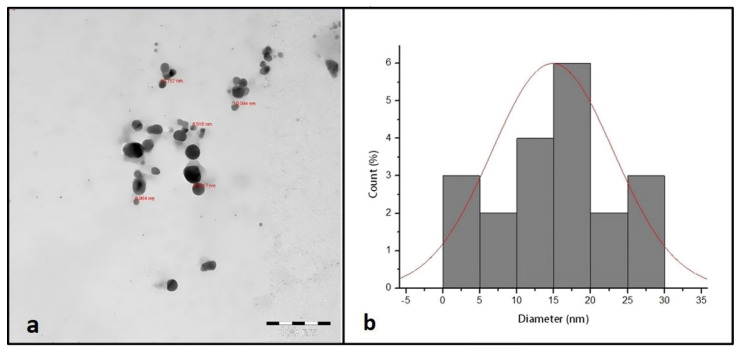
TEM image of synthesized AgNPs (**a**). TEM size distribution of synthesized AgNPs (**b**).

Figure 2a shows the TEM micrograph of synthesized AgNPs. The TEM image illustrated that the synthesized AgNPs were spherical in shape with a narrow size distribution ranging between 9 and 30 nm. 

### 3.2. Plant Growth Traits and Biomass Yield

**Figure 3:**
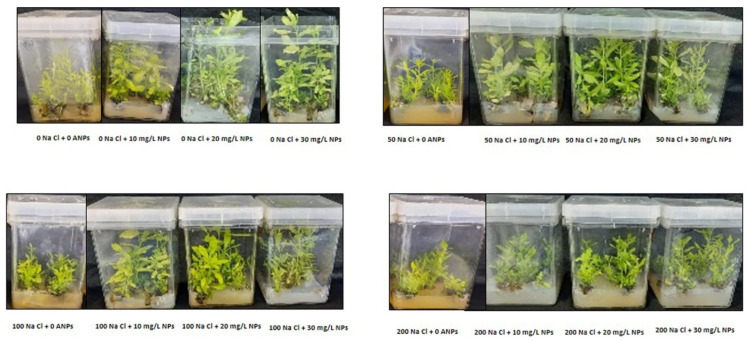
Shoots of *Maerua oblongifolia* plants under NaCl stress and application of biosynthesized silver nanoparticles.

The salt stress treatment (50, 100, and 200 mM NaCl) significantly lessened the fresh weight, dry weight, length of shoots, and shoot number in comparison with control plants. However, the supply of AgNPs significantly restored these mentioned morphological parameters (Figure 3). A great improvement was observed with the application of 10, 20, and 30 mg/L AgNPs against 50 and 100 mM NaCl treatments. While at 200 mM NaCl exposed plants, the AgNPs treatments showed a slight increase in fresh weight, dry weight, length of shoots, and shoot number (Table 1).

### 3.3. Photosynthetic Pigments

**Figure 4:**
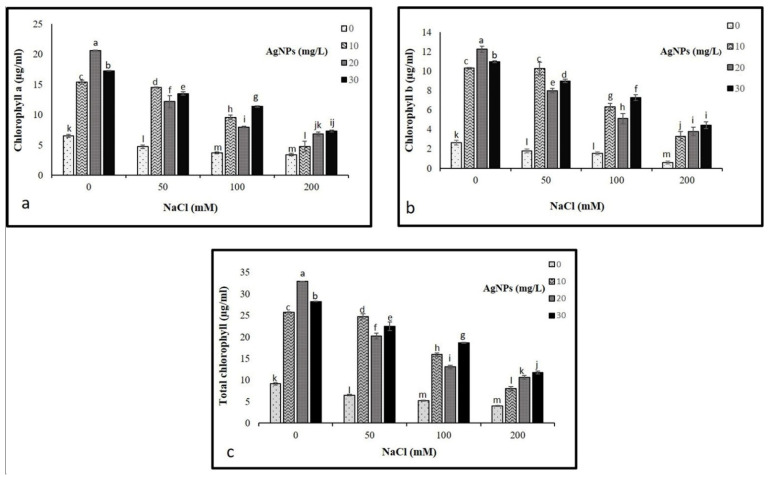
Photosynthetic pigments (**a**) Ch a, (**b**) Ch b, and (**c**) total chlorophyll in *Maerua oblongifolia* leaves under salt stress and application of silver nanoparticles. Means ± SD for each treatment followed by the same letters are not significantly different according to the Duncan’s test (*p* ≤ 0.05).

The findings showed a drastic decline in chlorophyll a and chlorophyll b at 50, 100, and 200 mM NaCl compared to control. Where the highest decrease was recorded in plants treated with 200 mM NaCL. Interestingly, the application of AgNPs with their all concentrations (10, 20, and 30 mg/L) enhanced the pigments contents in salinized and non-salinized plants, particularly at 50 mM NaCl driving a remarkable increase in chlorophyll a and b as depicted in (Figure 4). The AgNPs at 30 mg/L resulted in the greatest increase in pigment content when employed against the higher levels of salinity (100 and 200 mM). While 10 mg/L of AgNPs with 50 mM NaCl was most effective in enhancing the level of chlorophyll pigments.

### 3.4. Proline and Soluble Sugar Contents 

**Figure 5:**
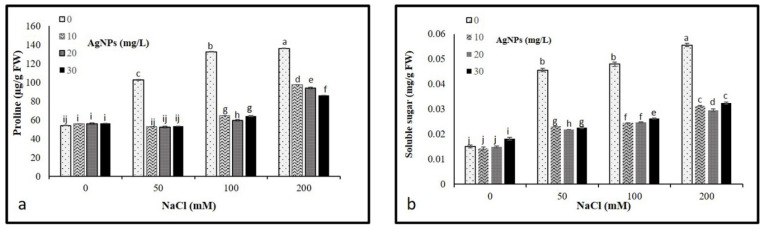
Osmolytes contents (**a**) proline and (**b**) total sugars in *Maerua oblongifolia* leaves under salt stress and application of silver nanoparticles. Means ± SD for each treatment followed by the same letters are not significantly different according to the Duncan’s test (*p* ≤ 0.05).

Our results revealed a significant increase in osmolytes (proline and soluble sugars) with an increase in salt concentration compared to control. The highest soluble sugars and proline contents were recorded in the plants under 200 mM salt without AgNPs application, whereas control plants had the lowest content. However, the application of AgNPs decreased the leaf accumulation of total sugars and proline in salt-stressed plants. Interestingly, all AgNPs concentrations (10, 20, and 30 mg/L) demonstrated their effectiveness in reducing osmolyte contents in salinized plants, the highest reduction was recorded against 50 mM NaCl (Figure 5a,b).

### 3.5. Lipid Peroxidation and Hydrogen Peroxide Contents

**Figure 6:**
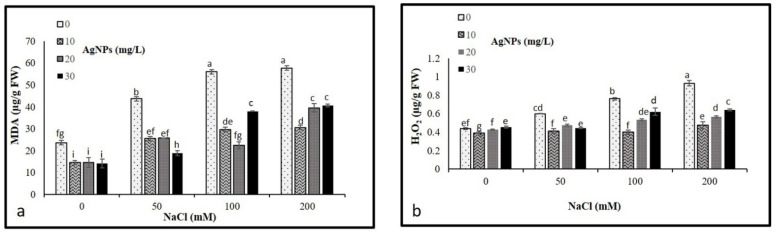
Contents of (**a**) MDA and (**b**) H_2_O_2_ in *Maerua oblongifolia* leaves under salt stress and application of silver nanoparticles. Means ± SD for each treatment followed by the same letters are not significantly different according to the Duncan’s test (*p* ≤ 0.05).

The levels of malondialdehyde (MDA) and hydrogen peroxide (H_2_O_2_) concentrations were analyzed to determine their possible oxidative damage in plants. As per our result, NaCl treatments resulted in a significant accumulation in MDA and H_2_O_2_ levels, where a remarkable rise in their contents was observed by increasing the levels of salinity. In total, 200 mM salt without AgNPs application recorded the maximum level of MDA and H_2_O_2_ (Figure 6a,b). However, levels of both MDA and H_2_O_2_ were diminished in the stressed and non-stressed *M. oblongifolia* plants after treatment with AgNPs. The lowest level of MDA and H_2_O was recorded at only the AgNPs treatments. 

### 3.6. Antioxidant Enzyme Activities

**Figure 7:**
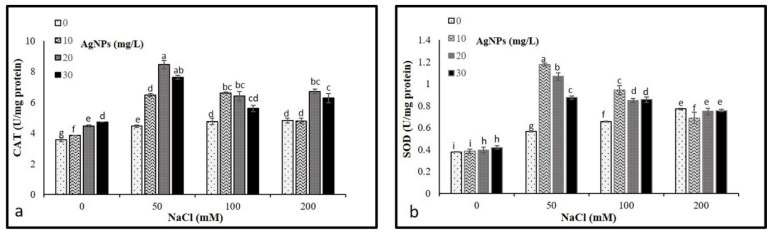
Activities of antioxidant enzymes (**a**) CAT and (**b**) in *Maerua oblongifolia* leaves under salt stress and application of silver nanoparticles. Means ± SD for each treatment followed by the same letters are not significantly different according to the Duncan’s test (*p* ≤ 0.05).

The activities of CAT and SOD were analyzed to determine their levels on the *M. oblongifoli* shoots under salt conditions with the presence of biosynthesized Nano-silver. The results revealed a significant increase in CAT and SOD activity compared to control. Interestingly, the application of AgNPs in stressed and non-stressed further raised the activity of both enzymes. The application of 20 mg/L AgNPs to plants treated with 50, 100, and 200 mM NaCl showed higher CAT activities compared to other 10 and 30 mg/L AgNPs concentrations (Figure 7a). While 10 and 20 mg/L, AgNPs with 50 mM NaCl were recorded as the highest levels of SOD (Figure 7b).

### 3.7. Nonenzymatic Antioxidants

**Figure 8:**
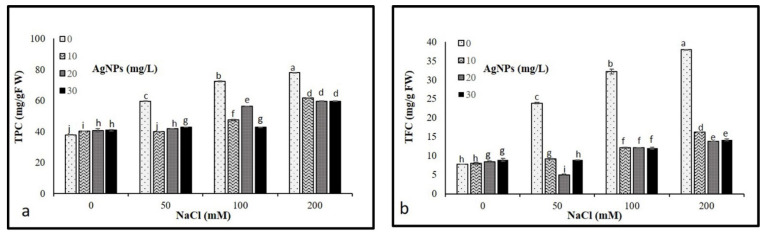
Non- Non-enzymatic antioxidant compounds (**a**) TPC and (**b**) TFC in *Maerua oblongifolia* leaves under salt stress and application of silver nanoparticles. Means ± SD for each treatment followed by the same letters are not significantly different according to the Duncan’s test (*p* ≤ 0.05).

Results illustrated in Figure 8 show that the progressive increase in salt concentrations (50, 100, and 200 mM) resulted in a significant increase in total phenolic and flavonoid compounds. However, the application of AgNPs caused a pronounced decrease in TPC and TFC in stressed and non-stressed *M. oblongifolia* shoots. Where AgNPs with 50 mM NaCl recorded the lowest levels of both TPC and TFC.

## 4. Discussion

Characterization of synthesized AgNPs is commonly performed using UV, XRD, FTIR spectroscopy, and TEM microscopy. These techniques provide information on the formation, size, structure, and elemental composition of nanoparticles. In this study, the fabrication of AgNPs was confirmed visibly by changing the color of the reaction mixture from faint yellow to brown after heating. It could be attributed to the existence of flavonoids and phenols in the leaf extract that are held responsible for the biosynthesis of AgNPs from Ag^+^. This result of color changes was in agreement with a previous study reported by Khan et al. [19]. UV-Vis spectroscopy showed a unique SPR absorption band at around 400 nm, typical in AgNPs, and indicated that the particles were dispersed without aggregation [20]. The XRD pattern of AgNPs confirms the crystallinity with a face-centered cubic (FCC) plane ((JCPDS, File No. 4-0787), and corresponds to pure Ag metal with FCC symmetry. Bearing in mind the FWHM of the plane in (111), the average particle size of synthesized AgNPs was found to be in the order of 16 nm. The FT-IR spectrum results showed an absorption peak at 3440 cm^−1^, potentially resulting from OH stretching the band at 1634 cm^−1^, assigned to the amide group, appears to be caused by carbonyl stretching in proteins [21]. The peak at around 800 cm^−1^ corresponded to the C-H bending of the monosubstituted alkene group, and those at around 675 cm^−1^ were assigned to the CH alkynes group [22]. Using *O. arabicus* leaf extract, Ag+ was reduced to create AgNPs, which were then characterized by TEM for their size and morphology. The TEM photographs clearly demonstrated that the synthesized AgNPs were spherical. This result is in agreement with previous results obtained by [23,24]. The dispersion characteristics of the spherical particles can differ according to their composition, size, and shape [25].

Exposure to green synthesized AgNPs has played an efficacious role in alleviating salt stress on different plant species and improving the plant growth profile [26]. The outcomes of the present study showed that salt stress significantly decreased plant growth by lowering the developmental characteristics of *M. oblongifolia*. Supporting this finding, several previous studies reported that salt stress lessened plant development and biomass in many plant species, including *Oenanthe javanica* [27] and *Sapium sebiferum* [28]. The lowering in plant growth under salinity is most likely attributed to the insufficient nutrient uptake by plants or higher translocation of Na to the shoots [29,30]. Furthermore, the remarkable decrease in the agronomic parameters of the plant under slat treatment indicated the high toxicity effects of salt stress on *M. oblongifolia* shoots. Interestingly, the application of AgNPs demonstrated positive effects on the developmental traits under salt treatments and significantly mitigated the stressor’s negative effects. Similarly, the application of AgNPs enhanced plant growth and biomass as well as increased plant tolerance to high salinity on different plant species such as *Satureja hortensis* [9]. The enhancement in growth parameters of *M. oblongifolia* shoots under salt stress after exposure to AgNPs in this study is most probably associated with enhancing the supply of various nutrient elements to plants [31], an increase in photosynthesis pigment level, and the mediating role of AgNPs in mitigation of salt stress [32].

The results of the present study showed that salt stress caused a significant decrease in chlorophyll a and chlorophyll b contents in *M. oblongifolia* shoots. The significant diminution in photosynthetic pigment content is most likely attributed to an increase in chloroplast structure damage or oxidative stress in plants [33]. Another possible reason for the excessive reduction in chlorophyll pigments might be the squandering of a major proportion of light energy as heat under salt stress [34]. A similar decrease in photosynthetic component parameters under salinity was reported earlier in maize [35], as well as in sorghum [36]. Salt stress has been reported to inhibit plant growth by causing oxidative stress, lessening chlorophyll content, reducing iron uptake and its translocation to the leaves, and inhibiting stomatal conductance [37,38]. However, the application of AgNPs improved the concentrations of chlorophyll a, b, and total chlorophyll in *M. oblongifolia*. Corresponding to our findings, the positive impacts of AgNPs on enhancing chlorophyll pigments under stressful conditions have been reported in *Linum usitatissimum*. The increase in the examined photosynthetic pigment contents after AgNPs application is most likely attributed to the enhancement in light energy of photosystem I (PSI) absorbed by the chloroplast membrane to be conveyed to photosystem (PSII) [39]. Furthermore, in a study reported by [40], he observed that the supply of AgNPs enhanced the chlorophyll content and facilitated the absorption of nitrogen and phosphorus through xylems in *Phaseolus vulgaris*. Therefore, the supplementation of Ag nanoparticles increased *M. oblongifolia* growth under saline toxicity by recovering chlorophyll content and improving the performance of the photosynthetic apparatus.

Plants naturally increase the production of soluble sugar and proline content in the cytosol and other organelles to cope with the reverse effect of salt toxicity that causes superfluous osmotic stress [41]. In our study, an increase in proline and soluble sugar content was observed in *M. oblongifolia* plants exposed to salt stress. However, the supply of AgNPs to salt-stressed *M. oblongifolia* plants effectively lessened the production of proline and soluble sugars. Our results are similar to the data obtained by Sultana, et al. [42], who reported that AgNPs reduced proline and soluble sugar content in rice under salinity stress.

The superfluous accumulation of salt in the cytoplasm causes hyperosmotic stress and ionic imbalance, hence, inducing ROS such as H_2_O_2_ as well as causing lipid peroxidation, which could result in great damage to the photosynthetic pigments, lipids, nucleic acid proteins, and cell membranes [43,44]. In this study, salt stress enhanced the levels of H_2_O_2_ and MDA, indicating the induction of oxidative damage in *M. oblongifolia* shoots. The high elevation of MDA and H_2_O_2_ is most likely due to the demolition of the cellular membrane integrity and cellular contents such as proteins and lipids [45]. Similarly, several studies reported that the content of H_2_O_2_ and MDA was drastically enhanced under salinity in different plant species, such as mung bean [46] and maize [47]. However, the findings of this study showed the obvious protective role of AgNPs against oxidative stress, where AgNPs considerably mitigated the H_2_O_2_ and MDA content in *M. oblongifolia* shoots treated with NaCl, thus improving the tolerance of a plant to injury normally induced by salinity stress. The role of AgNPs in improving plant tolerance to NaCl stress by reducing oxidative damage was also reported in pearl millet [48]. The reason behind the significant decrease in H_2_O_2_ and MDA is that AgNPs have the ability to upregulate the antioxidant system by speedy disposal of H_2_O_2_, therefore encouraging and maintaining plant growth [49].

Plants have a devoted detoxification system for scavenging ROS levels, which is constituted by various enzymatic and non-enzymatic antioxidants [50]. CAT and SOD represent the utmost common enzymatic antioxidants in plants. They function as a protection system in stress-induced responses [51]. Escalating the enzymatic antioxidant activities and developing antioxidant metabolism is one of the essential ways to improve salinity tolerance in plants [52]. In our study, the activities of CAT and SOD were increased in *M. oblongifolia* shoots under salt stress compared to controls. In correspondence to the findings of this study, the enzyme activity of SOD and CAT increased in *Solanum lycopersicum* L., *Zea mays*, *Brassica juncea,* and *Pistachio vera* in response to a progressive elevation in NaCl [53]. Interestingly, the application of 20 and 30 mg/L AgNPs to *M. oblongifolia* shoots treated with 50 mg NaCl showed a greater increase in enzyme activity of SOD and CAT compared to only salt-stressed plants. The enhanced antioxidant enzymes are most likely attributed to the active involvement of NPs in alleviating the toxic effect of stress [54]. Furthermore, the AgNPs application increased the antioxidant enzyme defense, which provided plants more strength to attain better regulation of increased ROS levels induced by salinity [55]. This finding is in agreement with results reported by Thuesombat et al. [56], who found an enhancement of antioxidant enzyme activities in rice exposed to AgNPs with the presence of salt stress. The improved activities of CAT and SOD by the application of AgNPs compared to only salt-stressed plants indicated that NPs may ameliorate the toxic effect induced by salt stress on the plants [48].

Phenolic and flavonoid compounds as non-enzymatic play a critical role in defending the plants from damaging effects brought on by numerous biotic and abiotic stressors as well as serving as cofactors for enzymes, affecting plant boom and its development from the initial development phase’s senescence [57]. In this investigation, salt-stressed *M. oblongifolia* shoots enhanced the production of total phenolics and flavonoids. Increased accumulation of total phenolic and flavonoid levels in response to salt stress was reported in wheat [58,59]. Application of AgNPs to *M. oblongifolia* plants stabilized the accumulated levels of total phenol and flavonoid content in the plant. They increased the capability to assist the plants to cope with oxidative damage caused by abiotic stress conditions [60]. The finding is in agreement with the observations of Khan et al. [61] who reported phenolic and flavonoid compounds decreased in the response to AgNPs under salinity stress. 

The results of this study indicated that the harmful effects of salt stress could be minimized by applications of AgNPs; therefore, the biosynthesized AgNPs can be applied in salinized agricultural farming as an ecofriendly, inexpensive, and bio-inoculant to enhance crop productivity.

## 5. Conclusions

In conclusion, the experiments revealed that the application of AgNPs could be an effective approach for the successful tolerance of *M. oblongifolia* plants under saline conditions. AgNPs applied to shoots of *M. oblongifolia* have induced significant changes in plants with respect to salt applications. AgNPs at 20 and 30 mg/L promoted the growth attributes and regulated enzymatic and non-enzymatic antioxidants. While significantly reducing the oxidative damage induced to plants by minimizing the accumulation of H_2_O_2_, MDA, and proline content. Overall, our findings suggest that the AgNPs can be a beneficial, ecofriendly application to reinforce the plant’s toleration against salt stress. However, molecular and genetic studies are required to understand the AgNPs-based salt tolerance mechanism in plants.

## Figures and Tables

**Table 1:** Effect of silver nanoparticles on the in vitro regeneration of *Maerua oblongifolia* exposed to salt stress after 45 days of treatment in MS media.

NaCl × AgNPs		Fresh Weight (g)	Dry Weight (g)	Shoot Number (pot)	Shoot Length (cm)
**0 NaCl**	**0 mg/L NPs**	7.06 ± 0.15 ^j^	1.40 ± 0.17 ^e^	11.66 ± 0.57 ^g^	6.46 ± 0.05 ^h^
	**10 mg/L NPs**	16.9 ± 0.10 ^c^	3.2 ± 0.20 ^c^	20.5 ± 0.20 ^b^	13.3 ± 0.15 ^b^
	**20 mg/L NPs**	18.2 ± 0.15 ^a^	4.3 ± 0.15 ^a^	21.2 ± 0.11 ^a^	14.2 ± 0.15 ^a^
	**30 mg/L NPs**	17.2 ± 0.15 ^b^	4.1 ± 0.20 ^a^	21.4 ± 0.30 ^a^	13.8 ± 0.10 ^b^
**50 NaCl**	**0 mg/L NPs**	6.30 ± 0.20 ^k^	1.06 ± 0.05 ^f^	10.66 ± 0.57 ^h^	5.93 ± 0.10 ^i^
	**10 mg/L NPs**	15.3 ± 0.10 ^de^	3.73 ± 0.05 ^b^	20.66 ± 0.57 ^b^	12.73 ± 0.05 ^c^
	**20 mg/L NPs**	15.53 ± 0.30 ^d^	3.63 ± 0.10 ^b^	20.66 ± 0.57 ^b^	12.87 ± 0.05 ^c^
	**30 mg/L NPs**	14.86 ± 0.15 ^e^	3.36 ± 0.05 ^c^	18.33 ± 0.57 ^d^	12.43 ± 0.05 ^cd^
**100 NaCl**	**0 mg/L NPs**	5.66 ± 0.15 ^l^	0.90 ± 0.11 ^fg^	10.33 ± 0.57 ^h^	4.1 ± 0.05 ^h^
	**10 mg/L NPs**	14.76 ± 0.15 ^e^	3.33 ± 0.10 ^c^	19.33 ± 0.57 ^c^	11.23 ± 0.05 ^e^
	**20 mg/L NPs**	15.3 ± 0.05 ^de^	3.70 ± 0.10 ^b^	20.66 ± 0.57 ^b^	12.63 ± 0.05 ^c^
	**30 mg/L NPs**	14.23 ± 0.05 ^f^	3.10 ± 0.23 ^c^	18.33 ± 0.57 ^d^	12.46 ± 0.05 ^cd^
**200 NaCl**	**0 mg/L NPs**	5.03 ± 0.15 ^m^	0.73 ± 0.05 ^g^	9.33 ± 0.57 ^i^	3.86 ± 0.05 ^j^
	**10 mg/L NPs**	7.96 ± 0.15 ^i^	1.43 ± 0.15 ^e^	11.33 ± 0.57 ^f^	6.96 ± 0.05 ^h^
	**20 mg/L NPs**	8.53 ± 0.15 ^h^	1.8 ± 0.10 ^d^	11.66 ± 0.57 ^f^	8.433 ± 0.05 ^g^
	**30 mg/L NPs**	11.06 ± 0.25 ^g^	1.96 ± 0.05 ^d^	15.33 ± 0.75 ^e^	9.267 ± 0.11 ^f^

The data representing the mean values of triplicates with ± standard deviation within a column followed by the same letters are not significantly different according to one-way analysis of variance (*p* ≤ 0.05).
